# Age-Related Functional and Expressional Changes in Efflux Pathways at the Blood-Brain Barrier

**DOI:** 10.3389/fnagi.2019.00196

**Published:** 2019-07-30

**Authors:** Franciska Erdő, Péter Krajcsi

**Affiliations:** ^1^Faculty of Information Technology and Bionics, Pázmány Péter Catholic University, Budapest, Hungary; ^2^Solvo Biotechnology, A Charles River Company, Budapest, Hungary; ^3^Faculty of Health Sciences, Semmelweis University, Budapest, Hungary

**Keywords:** blood-brain barrier, aging, efflux transporters, P-glycoprotein, BCRP, Alzheimer’s disease, Parkinson’s disease, stroke

## Abstract

During the last decade, several articles have reported a relationship between advanced age and changes in the integrity of the blood-brain barrier (BBB). These changes were manifested not only in the morphology and structure of the cerebral microvessels but also in the expression and function of the transporter proteins in the luminal and basolateral surfaces of the capillary endothelial cells. Age-associated downregulation of the efflux pumps ATP-binding cassette transporters (ABC transporters) resulted in increased permeability and greater brain exposure to different xenobiotics and their possible toxicity. In age-related neurodegenerative pathologies like Alzheimer’s disease (AD), the amyloid-β (Aβ) clearance decreased due to P-glycoprotein (P-gp) dysfunction, leading to higher brain exposure. In stroke, however, an enhanced P-gp function was reported in the cerebral capillaries, making it even more difficult to perform effective neuroprotective therapy in the infarcted brain area. This mini-review article focuses on the efflux functions of the transporters and receptors of the BBB in age-related brain pathologies and also in healthy aging.

## Introduction

**Figure 1:**
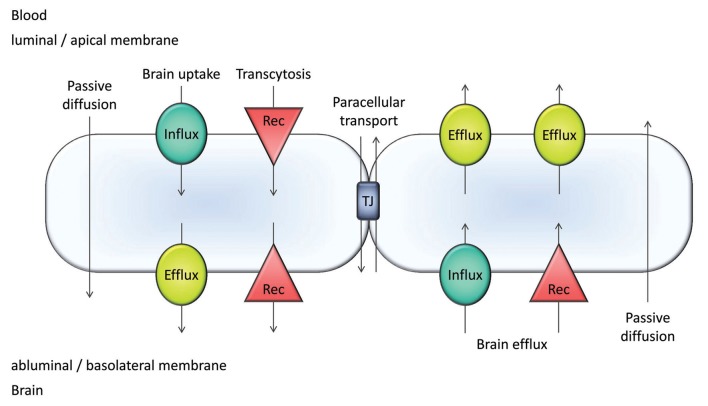
Major transport pathways at the blood-brain barrier (BBB). Transcellular transport occurs by passive diffusion or is mediated by influx and efflux transporters/carriers and/or receptors (Rec). Passive diffusion can be bidirectional; however, it is most often a blood-to-brain transport utilized by intermediate/high passive-permeability drugs. Paracellular transport is only significant for small compounds (MW <250 Da).

The blood-brain barrier (BBB) is a defensive structure and mechanism of the cerebral capillaries which works against chemical and microbial agents which could endanger the finely-regulated brain homeostasis (Erdő et al., 2017). The most important elements of the BBB are the capillary endothelial cells, which are connected by tight junctions (TJs) and adherens junctions (AJ) and surrounded by the basal membrane (Ceafalan et al., 2019), pericytes, and astrocyte endfeet. The paracellular transport of xenobiotics is blocked by occludin, claudin, and junctional adhesion molecule (JAM) proteins, connected to zonula occludens (ZO) proteins 1, 2, and 3, respectively. The AJ proteins (nectin and cadherins) also play an important role in the blockade of intercellular transport. The efflux transporter proteins expressed on the luminal surface of endothelial cells (Figure 1) amplify the protection provided by the compact cellular layers of brain microvessels and regulates the transcellular transport. These proteins transport their substrates against the concentration gradient back into the cerebral circulation and this way they can protect the brain parenchyma from dangerous molecules.

The BBB mediates a bidirectional transport of molecules (Figure 1). For small molecules, the major players are the transporters/carriers. For larger molecules such as peptides, receptor mediated pathways also contribute to transport (Figure 1).

This process is affected by aging. Both the expression level and the functionality of ATP binding cassette transporter proteins (ABC transporters) go through a transformation in aged subjects under both physiological and pathological conditions. The data in the literature available on these transformations are briefly summarized in this article. Paracellular permeability is also affected by physiological and pathological aging. Since it has been covered in detail in recent reviews (Storck et al., 2018), however, it is not covered in the present review article.

## Efflux Functions in Brain Microvessels

In Abcb1a/Mdr1a knockout mice, brain concentration of Mdr1a substrate ivermectin increased 87-fold, while plasma concentration increased only 3.3-fold (Schinkel et al., 1994). This increase of ivermectin brain exposure showed the pivotal importance of Abcb1a in the BBB and was in good agreement with the 50–100-fold increase in neurotoxicity of the drug (Schinkel et al., 1994). ABCB1/Abcb1a is localized in the luminal membrane of the BBB endothelial cells (Roberts et al., 2008). As it displays a broad substrate specificity, it provides significant protection to the brain.

ABCG2/BCRP/Abcg2/Bcrp1 is another luminally localized efflux transporter thought to play a major role in protecting the brain against toxicants. ABCG2/Abcg2 and ABCB1/Abcb1a have overlapping substrate specificities and thus may provide dual protection against toxicants, as shown by studies utilizing knockout mice (Agarwal et al., 2011). In double knockouts brain exposure to a substrate drug has often been found >10-fold greater than brain exposure in wild type animals, and a synergistic effect has been suggested. A detailed kinetic analysis of Abcb1a and Abcg2 function has shown that double knockouts over-predict drug-induced inhibition of both transporters, as clinically relevant concentrations of perpetrators are not likely to yield complete inhibition of ABCB1 and ABCG2 (Kalvass et al., 2013). Complete consensus on the potential risk of ABCB1 and ABCG2-mediated drug-drug interaction at the BBB has still not been reached, as it has been claimed that for victim drugs (such as several antivirals with high-fraction transported values), a potential for a clinically significant increase in brain exposure may occur (Prasad and Unadkat, 2015). It is also of note that ABCB1 inhibition yielded greater increases in brain distribution of dual substrate [^11^C]tariquidar in patients carrying one copy of the functionally impaired allele (c.421CA genotypes) of ABCG2 than in wild-type patients (c.421CC genotype; Bauer et al., 2016b). ABCB1 and ABCG2 cover a broad substrate specificity range. In addition to hydrophobic substrates, ABCB1 transports cationic drugs and ABCG2 transports many acidic drugs (Sedykh et al., 2013).

ABCC4/MRP4/Abcc4/Mrp4 is the third major efflux transporter expressed in the BBB (Table 1). Localization of ABCC4 is somewhat ambiguous as it was shown to be expressed in both the luminal and abluminal membranes of the BBB endothelium of the bovine brain microcapillary endothelium (Zhang et al., 2004). In contrast, in humans, it has only been detected in the luminal membrane (Nies et al., 2004). ABCC4 preferentially transports amphiphilic anions such as prostaglandins, steroid and bile salt metabolites, and drugs such as Ro-64-0802 the pharmacologically active form of oseltamivir (Ose et al., 2009).

**Table 1:** Absolute proteomics data of blood-brain barrier (BBB) efflux functions covered by this review*.

		Rat			Mouse		Monkey			Canine
Transporter/receptor	Localization	Human			Wistar	SD**	SD**	C57BL6	FVB	Indonesian cyno	Chinese cyno	Marmoset	
ABCB1	Luminal	3.98 ± 0.88	6.06 ± 1.69	2.58 ± 0.93	19.2 ± 2.0	19.0 ± 1.1	23.8 ± 0.32	1.22 ± 0.10	16.3 ± 0.8	2.65 ± 0.12	5.12 ± 0.91	6.48 ± 1.31	6.2 ± 1.39
ABCC4	Luminal and abluminal	0.31 ± 0.11	0.195 ± 0.069		1.40 ± 0.08	1.60 ± 0.029			2.18 ± 0.13	0.201 ± 0.041	0.303 ± 0.008	0.32 ± 0.057	ULQ
ABCG2	Luminal	6.15 ± 1.41	8.14 ± 2.26	2.22 ± 0.61	5.74 ± 0.50	4.15 ± 0.29	0.854 ± 0.00	ULQ	3.53 ± 0.21	14.1 ± 0.3	14.2 ± 1.4	16.5 ± 1.4	40.4 ± 8.4
LRP-1	Abluminal	1.76 ± 0.76	1.51 ± 0.26		1.09 ± 0.14	1.09 ± 0.14			1.02 ± 0.20		1.29 ± 0.05***		1.43 ± 0.64
		Shawahna et al. (2011)	Uchida et al. (2011b)	Al-Majdoub et al. (2019)	Hoshi et al. (2013)	Hoshi et al. (2013)	Gomez-Zepeda et al. (2019)	Gomez-Zepeda et al. (2019)	Agarwal et al. (2012)	Ito et al. (2011)	Ito et al. (2011)	Hoshi et al. (2013)	Braun et al. (2017)

*fmol/μg protein; **Sprague–Dawley; ***average of all monkeys.

Quantifiable expression of ABCA2 and ABCA8 in the BBB endothelial cells has been also reported (Table 1). ABCA2 is an endolysosomal protein and plays a pivotal role in the homeostasis of various lipids (Davis and Tew, 2018). As such, it has been linked to Alzheimer’s disease (AD; Davis, 2015). ABCA8 is also a lipid transporter and has been implicated in various brain pathologies (Jha et al., 2019). The location of ABCA8 in the BBB is not known. It is sinusoidally localized in hepatocytes (Sasaki et al., 2018).

Lipoprotein receptor-related protein-1 (LRP-1) also contributes to brain efflux of amyloid-β (Aβ) peptide (Shibata et al., 2000). Therefore, it is often discussed along with ABCB1 with regard AD and aging (Osgood et al., 2017). A strong expression of the protein in the abluminal membrane has been reported in both rats (Osgood et al., 2017) and mice (Ma et al., 2018; Storck et al., 2018).

Among efflux transporter proteins quantified by proteomics, Abcb1a is the most abundant in rats and mice (Table 1). In monkeys as well as dogs, ABCG2/Abcg2 is significantly more abundant, and in humans, the expression of the two transporters is close to the same (Table 1). Therefore, it is likely that the dominant role of Abcb1a over Abcg2 shown in mice (Kodaira et al., 2010) would not directly translate into humans.

Small regional differences in transporter expression have also been observed. The human expression of ABCG2 and ABCB1 was about 40% greater in the occipital lobe than in the parietal lobe (Billington et al., 2019). In the canine brain, regional differences in Abcg2 protein expression levels were reported for the cerebellum and the brain stem (Braun et al., 2017).

The abundance of ABCB1 was lower in men than in women (Billington et al., 2019). This, however, did not lead to a statistically significantly greater brain distribution of ABCB1 substrate [^11^C]verapamil (Sasongko et al., 2005).

## Methods of Studying Efflux Transporter Expression and Function in the BBB

Early on, gene expression was studied at the mRNA level by qRT-PCR (Osgood et al., 2017; Bors et al., 2018). Expression testing by western blot was also common (Rosati et al., 2003; Osgood et al., 2017; Bors et al., 2018). Immunohistochemistry provides localization data and has been frequently used despite challenges in quantitation of the data. The contemporary approach is quantitative LC-MS/MS-based proteomics (Table 1).

To study efflux transporter function in rodents, microdialysis (Sziráki et al., 2011, 2013; Bors et al., 2018) and SPECT imaging (Bors et al., 2018) has been utilized. Quinidine is the typical ABCB1 probe for microdialysis studies (Sziráki et al., 2011, 2013; Bors et al., 2018), and applicability of (99mTechnetium)-2-methoxy-isobutyl-isonitrile (MIBI) as an ABCB1 probe in SPECT studies has been demonstrated (Bors et al., 2018).

With the advent of position-emission computed tomography (PET-CT), functional testing of efflux transporters in the human BBB has become common (Toornvliet et al., 2006; Bartels et al., 2009; Bauer et al., 2009; Syvänen and Eriksson, 2013; Liow et al., 2016). [^11^C]Verapamil has been the ABCB1/P-glycoprotein (P-gp) selective probe substrate most commonly used in the studies and the applicability of [^11^C]Loperamide as well as [^11^C]Desmethylloperamide has also been demonstrated (Syvänen and Eriksson, 2013). Probes such as [^11^C]Elacridar and [^11^C]Tariquidar are dual substrates of ABCB1 and ABCG2 (Syvänen and Eriksson, 2013; Bauer et al., 2016a), and [^18^F]FCWAY is transported by ABCB1, ABCG2 and ABCC1 (Liow et al., 2016).

## Expression of Efflux Pathways Upon Physiological Aging

Expression and functional studies show a nice correlation between ABCB1 expression and function, as both show a decrease with physiological aging (Toornvliet et al., 2006; Bartels et al., 2009; Billington et al., 2019). Data on the effect of aging on expression of efflux transporters in humans has come from studies on postmortem tissue (Billington et al., 2019). Functional studies are more frequent, where PET-CT is used. One study in humans has shown gender specificity, as decreased function was seen only in men (van Assema et al., 2012). In dogs, a 72% drop in ABCB1 expression in animals older than 100 months over animals in the 23–36 month age group has been reported (Pekcec et al., 2011). A decrease in Abc1a expression and function in rats has also been reported (Rosati et al., 2003).

Data generated on SAMP8 in senescence-accelerated mice have shown increased expression of Abcb1a (Wu et al., 2009). However, data on BBB permeability is controversial in these animals, as increased permeability (Ueno et al., 1993; Pelegrí et al., 2007) and the lack of increase (Banks et al., 2000) have both been reported. Increase in Abcb1a expression would lead to an increased function that would be counterintuitive with increased permeability. As data in all species but mice have shown a decreased ABCB1 expression and function with age it is tempting to suggest that it is a general phenomenon across species and mice is not a good model for humans or accelerated aging does not correctly model all aspects of physiological aging.

ABCG2/BCRP is the other major efflux transporter luminally expressed in BBB endothelial cells, but no data have been reported on the effect of physiological aging on BCRP expression and/or function in the BBB. Changes in liver expression of ABCG2 upon aging have been studied, where no difference in mRNA levels but a decreased expression at the protein level has been shown (Riches et al., 2015).

In a rat model of aging, LRP1 expression on the basal surface of the BBB endothelium was robust in 3-month-old animals, but significantly reduced in 34-month-old animals (Osgood et al., 2017). A reduction in LRP1 mRNA levels in cerebral microvessels was also demonstrated in the same study.

## Mechanism of Regulation of Transporter Expression in Aging

The studies aiming to explore the mechanism of ABCB1 expression in humans have almost unanimously shown decreased protein expression and function in aging (see previous section for details). Levels of ABCB1 mRNA, however, have not always showed a decrease with age within the same study (Chiu et al., 2015).

In rats, the one study that covered Abcb1a expression at the level of both mRNA and protein found a parallel decrease in the two functions (Osgood et al., 2017). In the SAMP8 mice, Abcb1a mRNA levels increased more significantly than Abcb1a protein levels (Wu et al., 2009), but applicability of that model is questionable as this is the only model that shows an increased protein expression upon physiological aging (see previous section for details).

One study has addressed the mechanism of down-regulation of ABCB1. However, since patients with AD were also included in that study (Hartz et al., 2018), it is difficult to draw conclusions on the mechanism of the decrease of ABCB1 protein upon only physiological aging. In humans with AD, the levels of ubiquitination was greater than in age-matched cognitively normal humans (Hartz et al., 2018). Increased ubiquitination was also observed in transgenic human amyloid precursor protein (hAPP)-overexpressing mice (Tg2576) in the same study. It was also shown that PYR41, a cell-permeable, irreversible inhibitor of the ubiquitin-activating enzyme E1 inhibited ubiquitination, counteracted the decrease of Abcb1a and reduced Aβ plaque formation in the transgenic mice. Importantly, no decrease of LRP-1 activity in hAPP mice was observed (Hartz et al., 2018).

## Efflux Pumps at The Bbb in Age-Related Pathological Conditions

Aged people are at higher risk of medicine-induced toxicities resulting from either increased drug sensitivity or age-related pharmacokinetic changes. The situation is further complicated with the two most prevalent age-related neurodegenerative diseases, AD and Parkinson’s disease (PD), for both of which there is growing evidence of altered structure and function of the BBB, including downregulation of TJ proteins and efflux transporters such as P-gp (Bors et al., 2018). These alterations would have an impact on CNS drug exposure and the risk of neurotoxicity from systemically-acting drugs (Erdő et al., 2017; Pan and Nicolazzo, 2018). Qosa et al. (2016) have summarized the directions of the efflux transporter alterations at the BBB in different neurodegenerative disorders. They showed that P-gp is downregulated in AD, Creutzfeldt-Jakob syndrome, multiple sclerosis, brain tumors, PD, and schizophrenia, while it is upregulated in amyotrophic lateral sclerosis, epilepsy, stroke, and ischemic brain injuries. There are many unanswered questions about the role of BCRP, MRP1, and MRP2 in neurodegenerative diseases (Qosa et al., 2016). In the next paragraphs only the three most important examples are shown in more details.

### Alzheimer’s Disease

The involvement of transporters located at the BBB has been suggested in the control of cerebral Aβ levels, and thereby in AD. Active transport of Aβ across the BBB seems to involve a number of transporters that control the level of the soluble isoform of Aβ in brain. P-gp contributes to the efflux of brain-derived Aβ into blood (Kuhnke et al., 2007; Bell and Zlokovic, 2009; Hartz et al., 2010; Brenn et al., 2011; Vogelgesang et al., 2011; Sagare et al., 2012; Sharma et al., 2012; Erdő et al., 2017). It seems that, in addition to the age-related decrease of P-gp expression, Aβ1–42 itself downregulates the expression of P-gp and other Aβ transporters, which could exacerbate the intracerebral accumulation of Aβ and thereby accelerate neurodegeneration in AD and cerebral Aβ angiopathy. Systemic inflammation by lipopolysaccharide triggers defects in P-gp-mediated Aβ clearance from the brain and leads to its accumulation (Ravenstijn et al., 2008). Reduction of P-gp expression and transport activity has been found in isolated capillaries as a result of Aβ40 mediated P-gp ubiquitination, internalization, and proteasome-dependent degradation (Hartz et al., 2016). However, little is known about the regulation of these transporters at the BBB (Do et al., 2016) in animal models of AD. The most efficient therapeutic strategy for limiting the accumulation of Aβ in the brain as early as possible in disease development could be to block Aβ influx by inhibiting Rage and/or Oatp1a4, or to stimulate the synthesis and/or function of Abca1 and/or Abcg4 at the BBB (Do et al., 2016).

### Parkinson’s Disease

The brain distribution of compounds with different transport mechanisms across the BBB (l-3,4-dihydroxyphenylalanine, carbamazepine, quinidine, lovastatin, and simvastatin) in healthy and MPTP-treated macaques have been studied. Thiollier et al. (2016) found only changes in the distribution of quinidine, indicating changes in P-gp functionality. In contrast, studies performed by Hou et al. (2014) suggest that P-gp inhibition increases BBB permeability to N-[2-(4-hydroxy-phenyl)-ethyl]-2-(2,5-dimethoxyphenyl)-3-(3-methoxy-4-hydroxy-phenyl)-acrylamide (FLZ), a novel synthetic squamosamide derivative and potential anti-PD agent. No significant differences were observed, however, in the brain distribution of FLZ between normal and PD model rats, suggesting no significant change in P-gp in PD (Hou et al., 2014). Bartels et al. (2008a,b) investigated *in vivo* BBB P-gp function in patients with parkinsonian neurodegenerative syndromes using [^11^C]verapamil PET. Advanced PD patients had increased [^11^C]verapamil uptake in frontal white matter regions compared with controls. The authors concluded that lower [^11^C]verapamil uptake in midbrain and frontal regions of *de novo* PD patients could indicate a regional up-regulation of P-gp function (Bartels et al., 2008b). However, in a later study by this group, the decreased BBB P-gp function in early-stage PD patients could not be confirmed (Bartels et al., 2008a).

### Stroke

Stroke risk increases with aging, and one-third of ischemic strokes occur in the very elderly (> or = 80 years). These are responsible for two-thirds of the overall stroke-related morbi-mortality (Ly and Maquet, 2014).

Rats subjected to middle cerebral artery occlusion (MCAO) for 90 min and killed at 4, 14, 24, and 48 h postreperfusion onset were studied to determine the time course of P-gp expression. To mimic ischemia occurring at the BBB, rat brain endothelial (RBE4) cells were subjected to hypoxia and low glucose (HLG) for 16 h. Immunoblotting analyses showed P-gp increases in brain and liver following 90-min MCAO, as well as in cultured RBE4 cells after 16-h HLG treatment. The increase in P-gp could dramatically reduce the bioavailability and efficacy of neuroprotective drugs. P-gp, therefore, represents a big hurdle in drug delivery to the ischemic brain (DeMars et al., 2017).

## Conclusions and Outlook

Transporter proteomics has been used in the past decade to study expression in the BBB, but mostly from a perspective of drug exposure (Uchida et al., 2011a; Trapa et al., 2019), and many questions on the effect of aging and disease on efflux transporter expression and function are still open. The physiology and pathophysiology, as well as the cell biology and biochemistry of transport, are new fields. One uncertainty is the correlation of expression and activity of efflux proteins. In general, it is assumed that protein expression and activity for ABCB1 and ABCG2 correlate (Uchida et al., 2011a; Trapa et al., 2019). However, it is not always the case when mRNA levels and function have been monitored (Vilas-Boas et al., 2011). In some studies, discrepancies between protein expression and function have also been reported (Poller et al., 2010).

It is reasonable to assume that a complex interplay of age-related decrease and a disease-related stimulation shapes the expression profile of efflux transporters. Indeed, a biphasic regulation of ABCB1 in AD has been suggested (Vogelgesang et al., 2004). Also, ABCB1 and ABCG2 expression by neuroinflammatory stimuli displayed a complex pattern (von Wedel-Parlow et al., 2009). Inhibition of efflux function at the time when it plays a protective role is counterintuitive. In contrast, the inhibition of efflux functions to enhance the BBB permeability for drug delivery may make sense. The time dependence of pathophysiological changes must be pinned down to utilize the data to drive pharmaceutical and nutraceutical research.

In addition to temporal changes, regional variance (Deo et al., 2014; Kannan et al., 2017) in the expression of transporters is a factor to consider. Both could be monitored with imaging. However, [^11^C]verapamil is not an ideal probe for ABCB1, due to its lipophilicity (Luurtsema et al., 2016). Development of novel probes or employment of drug probes used for preclinical species could enable the scientific community to address these issues. ^18^F-FCWAY is a weak substrate of ABCB1 and has been suggested as a probe to address both up- and down-regulation of ABCB1 transport activities (Liow et al., 2016).

Therapeutic strategies must also take into account the fact that expression of proteins such as ABCA2 show an inverse correlation with disease (Michaki et al., 2012). Strategies based on the modulation of transporter expression must, therefore, use selective inducers/repressors to provide therapeutic efficacy. Induction of proteins that slow disease progression (e.g., ABCB1) without induction of proteins (e.g., ABCA2) that facilitate disease progression is one possible strategy. ABCB1 and ABCA2 display inverse regulation upon the differentiation of colon tumors (Ohtsuki et al., 2007). However, it remains to be tested if agonists of the pregnane X receptor (PXR) that induce ABCB1 expression have a favorable induction/repression profile on transporters and receptors also involved in pathogenesis of neurodegenerative diseases.

## Author Contributions

FE and PK contributed equally to this manuscript. Both authors conceived, revised, and approved the final manuscript.

## Conflict of Interest Statement

The authors declare that the research was conducted in the absence of any commercial or financial relationships that could be construed as a potential conflict of interest.
